# Perceived reporting-system function, reporting barriers, and formal near-miss reporting among operating room nurses in China: a cross-sectional study

**DOI:** 10.3389/fmed.2026.1997500

**Published:** 2026-10-13

**Authors:** PingPing Li, Lixue Zhang, Qin Cao, Xin Tan, Yanling Fan, Hui Hu

**Affiliations:** Department of Anesthesiology, Nanchong Central Hospital, Nanchong, Sichuan, China

**Keywords:** incident reporting, near miss, operating room nurses, patient safety culture, perioperative safety, speaking up

## Abstract

**Background:**

Near-miss reporting provides an early signal of perioperative system failure, yet formal reporting remains limited in operating rooms. This study examined correlates of formal near-miss reporting among operating room (OR) nurses at a two-campus tertiary hospital in China.

**Methods:**

In this cross-sectional study, 2,281 OR nurses returned a structured questionnaire and 2,200 were analyzed, covering self-reported 12-month near-miss observation and reporting, four safety-culture indices, workload, and professional characteristics. Exposures were purpose-built indices rather than validated scales. Multivariable logistic regression, with a prespecified parsimonious model as the principal specification, examined factors associated with formal reporting among observers (Model A) and, as an exploratory aim, near-miss observation in the full sample (Model B).

**Results:**

Of 2,200 OR nurses, 1,551 (70.5%) observed at least one perioperative near-miss during the preceding 12 months, yet only 478 (30.8%) formally reported an event; the remaining 1,073 (69.2%) made no report, a nurse-level rather than event-level measure. Count discrepancies predominated (47.3%). In the principal model, perceived reporting-system function was the strongest correlate of formal reporting (aOR: 2.19 per 1-SD increase, 95% CI: 1.84–2.60), whereas greater barrier burden was inversely associated (0.67, 0.57–0.79); speaking-up and safety climate, which overlapped substantially, were not independently associated. The reporting-system association persisted after removal of the feedback item, which may reflect earlier reporting, and ordinal modeling of reported event volume reproduced both associations. Model A discrimination was acceptable (AUC 0.741). In the full sample, higher time pressure was associated with near-miss observation (1.14, 1.03–1.25), but this exploratory model discriminated poorly (AUC 0.600).

**Conclusion:**

Perioperative near misses were commonly observed but seldom documented. These cross-sectional, self-reported associations cannot establish direction, but they are consistent with formal reporting depending on accessible, easy-to-use reporting systems and on reduced hierarchy-related and relational barriers, rather than on willingness to speak up alone.

## 1 Introduction

Surgical care is essential for managing diverse health conditions, such as injuries, obstructed labor, malignancy, infections, and cardiovascular disease, and is an indispensable component of a functioning health system (1, 2). In 2012, an estimated 312.9 million operations were performed worldwide, an increase over earlier global estimates (3, 4). In China, patient-safety culture in hospital and surgical settings remains an active improvement priority (5, 6). Despite the widespread implementation of safety checklists and structured protocols, the operating room (OR) remains one of the highest-risk environments in healthcare, where instrumentation, anesthesia, sterile field management, and patient identification occur simultaneously, with limited tolerance for error (7, 8).

A near-miss event is an unplanned sequence that could have harmed a patient but was prevented by chance or timely detection (9, 10). Near misses often arise from the same root causes as adverse events, but they occur more frequently and usually leave no injury record, making them an early signal of system vulnerability (9, 11). In the OR, these events commonly involve retained instruments, sponges, or needles; wrong-site procedures; medication or specimen labeling failures; and breaches of sterile techniques (12). Takahashi et al. reported near-miss events involving retained foreign bodies at 2.13 per 1,000 operations over ten years, with 30% identified because a scrub nurse detected a count discrepancy rather than through a formal safety system (13). Therefore, OR nurses frequently serve as the final protective barrier before latent errors reach the patients. However, formal near-miss reporting remains low in the field. Song et al. reported a medical error prevalence of 27.7% among Chinese OR nurses, with instrument-related events predominating (14). Wang et al. further showed that involvement in such errors was independently associated with greater psychological distress and suicidal ideation, indicating that the consequences extend beyond the immediate clinical risk (15). Despite this burden, national incident-reporting data and nurse surveys from China indicate that under-reporting is common, and many near misses are likely to be resolved informally or left undocumented (16, 17).

Barriers to voluntary reporting have been well established. Fear of blame is a major deterrent; Brabcová et al. found that the fear of being blamed for patient deterioration, followed by concerns about legal liability and punitive managerial responses, was the most strongly endorsed barrier to reporting among hospital nurses (18). In an eight-hospital Chinese study, Qin et al. similarly found that perceptions of managerial reactions, rather than knowledge deficits, were the strongest correlates of under-reporting, highlighting organizational leadership as a key target for improvement (19). Workload is a further plausible constraint: higher patient-to-nurse ratios are independently associated with poorer patient outcomes (20, 62), and in Chinese ORs, where nurses commonly manage several consecutive cases per shift, the time available to complete formal reports may be limited.

Speaking up about safety concerns is constrained by hierarchical and organizational factors. Schwappach and Richard found that a speak-up-related climate, including psychological safety and an encouraging environment, was associated with healthcare workers' speaking up and withholding voice (21). Behavioral studies in anesthesia extend this survey evidence by observing voice directly rather than through self-reported climate, and show that whether a concern is raised, how it is phrased, and whether it is acted upon are distinct and separately modifiable (22–26). A systematic review reported that more positive nurse safety attitudes were associated with better patient outcomes in acute-care hospitals (27). Similarly, Alzahrani et al. reported that teamwork climate and management support were among the lowest-scoring safety culture dimensions in hospital emergency departments and represented important intervention targets (28). These challenges may be more pronounced in Chinese hospitals, where hierarchical norms reinforce professional role boundaries (29). Zhu and colleagues found that error reporting was among the lowest-scoring safety-climate dimensions across six Chinese hospitals, with only 44.5% positive responses, and OR staff reported more negative perceptions than other staff groups (30). Although structured perioperative safety tools can improve outcomes when embedded within genuine team communication (31), effective teamwork may remain limited when professional groups work in parallel rather than through shared situational awareness (32).

Near-miss reporting can be viewed as a sequence: recognition of the event, real-time speaking up or intervention, the decision to file a formal report, and the organization's response and feedback, which may influence later reporting (11, 16). The constructs examined here sit at different points in this sequence: safety climate as the organizational context, speaking-up climate at real-time voice, and reporting barriers and reporting-system usability at the decision to report (18, 19, 21). Perceived feedback and the belief that reporting leads to change arise mainly after earlier reports, so they may be consequences as well as antecedents of reporting. Because these constructs overlap, mutually adjusted estimates are partial associations rather than separate effects.

Despite growing attention to patient safety culture in China, near-miss reporting has rarely been examined together with reporting-system function, speaking-up climate, reporting barriers, and safety climate among OR nurses. The present study addresses one transition within the sequence described above, from recognition of a near-miss to its formal documentation. It measures perceived climate and retrospectively self-reported behavior rather than observed voice, and therefore complements the behavioral literature on speaking up; because all measures came from one questionnaire, the associations may partly reflect common-method variance and cannot establish direction. Among OR nurses at a two-campus tertiary hospital in Nanchong, the study aimed to describe 12-month self-reported near-miss observation and the proportion of observers who made no formal report, a nurse-level rather than event-level measure; to identify factors associated with formal reporting among observers; and to explore factors associated with near-miss observation.

## 2 Methods

### 2.1 Study design and setting

This single-institution, two-campus cross-sectional questionnaire study was conducted among OR nurses at Nanchong Central Hospital, a Grade A tertiary teaching hospital with 4,200 beds in Nanchong, Sichuan 637000, China, between February 2026 and May 2026. The two campuses share a single perioperative nursing administration, a single incident-reporting platform, and one patient-safety office; nurses are centrally assigned and rotate between campuses, and the campuses are therefore treated as one source population rather than as independent centers. The perioperative service comprises 118 physical operating rooms (72 at Campus 1 and 46 at Campus 2) and performed approximately 142,000 operations in 2025. An operating suite is a physically contiguous block of operating rooms sharing a nursing station, sterile core, and head nurse; the 28 suites are grouped into 14 supervisory units. The study was designed and reported in accordance with the Strengthening the Reporting of Observational Studies in Epidemiology guidelines for cross-sectional studies (33).

A cross-sectional design was chosen to estimate the prevalence of self-reported near-miss observation and formal reporting over a 12-month recall window and to identify their correlates within one four-month enrollment period. As all exposures and outcomes were measured simultaneously using self-reports, the resulting associations were interpreted as adjusted epidemiological relationships rather than causal effects. The source population comprised all registered nurses assigned to OR services during the study period, encompassing circulating, scrubbing, specialty, and coordinating roles. Nurses were excluded if they were not engaged in OR clinical duties during the data collection window, if their questionnaire was missing the primary outcome response, if fewer than the minimum number of items required for any domain score was completed, or if the data quality review identified irreconcilable logical inconsistencies.

Recruitment ran for 16 weeks, from 9 February 2026 to 31 May 2026. Of the 2,918 registered nurses on the perioperative establishment on 9 February 2026 (1,777 at Campus 1 and 1,141 at Campus 2), 306 were not engaged in OR clinical duties (anesthesia recovery, sterile processing, or administrative posts) and were ineligible, leaving 2,612 eligible nurses who were invited. A total of 331 declined or did not respond, and 2,281 questionnaires were returned, giving a response rate of 87.3%. Eighty-one were excluded: 19 reassigned away from OR clinical duties during data collection, after they had been invited (through rotation, leave, or secondment), as confirmed against the duty roster; 34 for a missing primary outcome item; 12 for insufficient items to derive a domain score; and 16 for irreconcilable logical inconsistencies. The final analytic sample comprised 2,200 OR nurses, 1,340 (60.9%) from Campus 1 and 860 (39.1%) from Campus 2, proportions that correspond to the perioperative nursing establishment at each site (Figure 1).

**Figure 1:**
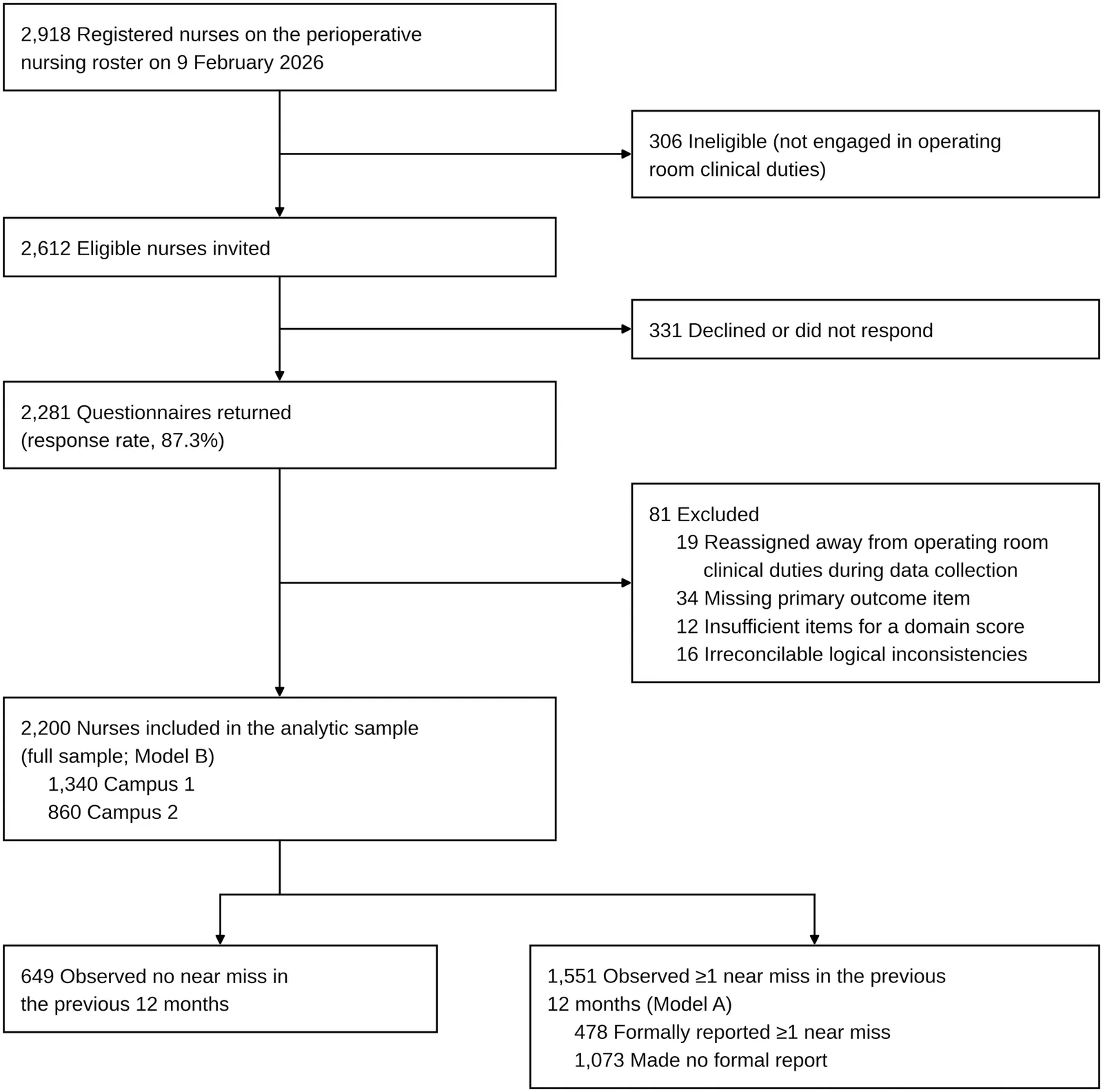
STROBE participant flow chart showing the flow of nurses from the perioperative nursing roster to the analytic samples for model B (full sample, *N* = 2,200) and model A (near-miss observers, *n* = 1,551).

The questionnaire was administered electronically through a secure institutional survey platform. Invitations were distributed by perioperative nursing administration through departmental communication channels on 9 February 2026, with two reminders at two-week intervals and closure of the platform on 31 May 2026. No incentives were offered, and participation had no bearing on performance appraisal. To prevent duplicate submission, each invited nurse received a single-use access link that expired on completion. The file linking tokens to invitees was held only by the hospital information-technology administrator, who had no study role, and was deleted at platform closure; reminders went to all invitees, and investigators and nursing administration received a de-identified export without names, employee numbers, tokens, or IP addresses, so responses could not be linked to individual nurses. Each nurse was invited once and contributed one questionnaire, so records are independent and the design is a single-wave cross-sectional survey. Questionnaires were completed by 486 respondents (22.1%) in February, 742 (33.7%) in March, 618 (28.1%) in April, and 354 (16.1%) in May 2026, with similar distributions at both campuses (all differences <4 percentage points) and no change in reporting infrastructure, nursing leadership, or safety policy during data collection.

### 2.2 Data collection and questionnaire instrument

Data were collected using a structured, self-administered questionnaire specifically developed for this study. Of its 20 domain items, 15 were adapted from published instruments, with reporting-system and safety-climate items drawn from the Hospital Survey on Patient Safety Culture and the Safety Attitudes Questionnaire (30, 34, 35), speaking-up items from the Speak Up-related Climate Scale (21), and barrier items from nurse reporting-barrier questionnaires (18, 19); the remaining 5 (questioning a surgeon, questioning an anesthesiologist, hesitation because of blame or conflict, an unclear near-miss definition, and near-miss reports improving workflow) were newly written for the OR context. Adapted items were translated from English into Mandarin, the language of development and administration, by two bilingual nurse researchers, and all items were back-translated by an independent translator blinded to the sources and reconciled by consensus, which refined two barrier items. Seven experts (two perioperative head nurses, two patient-safety officers, one anesthesiologist, one surgeon, and one nursing methodologist) rated item relevance on a four-point scale, giving item-level content validity indices of 0.86 to 1.00 and a scale-level average of 0.94 (36). Pilot cognitive interviewing with five non-study OR nurses then resolved remaining ambiguities; reported English stems reflect reconciled back-translations. Completed responses were entered into an electronic database with range, skip-logic, and consistency checks, and near-miss items specified a 12-month recall window.

The demographic variables included age group, sex, and highest level of nursing education. Occupational variables included professional title (five-tier national hierarchy: nurse, senior nurse, supervisor nurse, deputy chief nurse, and chief nurse); total nursing experience; OR-specific nursing experience; employment status; primary OR specialty assignment; average weekly working hours; night shifts per month; and surgical cases per working day. Both experience variables were retained as ordered categories, and prior safety training was coded as binary data. Four work context items measuring time pressure, staffing adequacy, routine preoperative briefings, and routine postoperative debriefings were analyzed separately from the domain scores as distinct OR process conditions. Time pressure and staffing adequacy were included in the regression models as standardized continuous variables. The four items were rated on the same five-point agreement scale: “I often work under time pressure during operations”; “Nurse staffing is adequate for the surgical workload”; “Preoperative team briefings are routine in my operating room”; and “Postoperative debriefings are routine in my operating room”.

### 2.3 Outcomes and operational definitions

A perioperative near-miss event was operationally defined as any unplanned circumstance within the OR that had the potential to cause patient harm but did not, whether through chance or active intervention, during the preceding 12 months. This definition was adapted from the World Health Organization's International Classification for Patient Safety and was presented in identical wording to every respondent to ensure a uniform frame of reference (10).

The primary outcome was formal near-miss reporting among nurses who had observed at least one event, coded as 1 if the respondent formally reported at least one near-miss, and 0 if they observed but did not report. The secondary outcome was near-miss observation in the entire sample, coded as 1 if the respondent observed at least one event, and 0 if none were observed. Observation was asked as “In the past 12 months, how many near misses have you personally observed in the operating room?” and reporting as “In the past 12 months, how many of these near misses did you formally report through the hospital incident-reporting system?”, each answered as none, 1, 2–5, 6–10, or more than 10. The proportion of observers who made no formal report was calculated with the count of observers reporting no event as the numerator and all near-miss observers as the denominator. This is a participant-level measure and does not represent the proportion of individual events that went unreported, because a respondent who observed several events and formally reported one was classified as a reporter. Observed and formally reported counts were recorded in the ordered bands 1, 2–5, 6–10, and >10 rather than as exact counts, so the event-level reporting fraction is not identifiable and was not estimated. Ordered-categorical (proportional-odds) modeling of reported event volume was prespecified as the complementary analysis.

Five near-miss subtypes were coded as binary indicators: patient identification or documentation errors; surgical site, procedure, or positioning events; instrument, sponge, or needle count discrepancies; medication, blood product, or specimen-labeling failures; and sterility, infection control, or equipment failures. Respondents could endorse more than one near-miss subtype. Subtype was recorded only for observed events; respondents were not asked which subtypes they had formally reported.

### 2.4 Exposure variables

Four safety culture domains were specified as the primary organizational exposures. Each was operationalized as the item-level mean of its constituent five-point Likert-scale responses, ranging from 1 (strongly disagree) to 5 (strongly agree), with no “not applicable” option. Internal consistency was evaluated using Cronbach's alpha and McDonald's omega, together with corrected item-total correlations (37), and Spearman's correlations were computed between domain pairs. The four-domain structure was additionally examined by confirmatory factor analysis using diagonally weighted least-squares estimation appropriate for ordered categorical indicators, with fit judged by the comparative fit index, Tucker–Lewis index, root mean square error of approximation, and standardized root mean square residual. Discriminant validity was assessed using the heterotrait-monotrait ratio of correlations, with values below 0.85 taken to indicate adequate discrimination (38).

Reporting-system function (alpha = 0.621) comprised “I know how to report a near miss”; “The reporting process is easy”; “I receive feedback after reporting a near miss”; and “Reporting near misses prevents future harm”. Respondents who had not reported were instructed to answer from what they knew of feedback on colleagues' reports. Because perceptions of feedback and preventive value may arise from a respondent's own earlier reports, the first two items formed a pre-report accessibility subscore and the last two a post-report experience subscore for sensitivity analysis. Speaking-up climate (alpha = 0.831) comprised “I feel comfortable speaking up about a safety risk”; “I feel comfortable questioning a surgeon”; “I feel comfortable questioning an anesthesiologist”; “Head nurses encourage staff to raise concerns”; “The team responds respectfully when I raise a concern”; and “I speak up to senior staff when patient safety is at risk”. Reporting barriers (alpha = 0.801) comprised “Fear of blame prevents me from reporting”; “Lack of time prevents me from reporting”; “I fear that reporting will damage my working relationships”; “I am concerned that reporting will affect my professional evaluation”; “I have hesitated to report because of blame or conflict”; “I believe nothing will change after reporting”; and “The definition of a near miss is unclear to me”, scored without reverse coding so that higher scores indicate greater deterrence; the “no change” item may also reflect earlier reporting experience. Safety climate (alpha = 0.706) comprised “Patient safety is a high priority in my unit”; “Near-miss reports improve our workflow”; and “Errors receive a fair, non-punitive response”.

Because the heterotrait-monotrait ratio for speaking-up and safety climate exceeded 0.85, and safety climate may act through speaking-up climate and reporting barriers, their mutually adjusted coefficients are interpreted as partial associations rather than independent effects; alternative specifications are described in Section 2.6. For all regression analyses, the four composite scores, together with time pressure and staffing adequacy, were standardized (mean 0, SD 1), so adjusted odds ratios represent the change in odds per 1-SD increment.

### 2.5 Data management and quality assurance

The dataset was systematically screened for duplicate records, out-of-range Likert values, missing primary outcome data, and structurally inconsistent response patterns before any analysis was performed. Logical checks confirmed that OR-specific experience did not exceed total nursing experience in any record, that age group and experience category combinations fell within plausible career timelines, and that near-miss subtype responses were absent among participants who reported observing no near-miss events. Domain scores were computed from all available items per respondent in accordance with the minimum-item rules (at least three of four items for reporting-system function, four of six for speaking-up climate, five of seven for reporting barriers, and all three for safety climate). Among the 2,200 analyzed respondents, item-level missingness ranged from 0.2% to 1.8% across the 20 domain items and was highest for the item on receipt of feedback after reporting (1.8%). At participant level, 187 respondents (8.5%) had at least one missing domain item but met the minimum-item rules. Because the feedback item may depend on reporting status, missingness is compared between reporters and non-reporters in Section 3.4, and a complete-case analysis restricted to observers with all 20 domain items was performed as a sensitivity analysis. No analyzed respondent had a missing composite score, and no further records were excluded from either regression model, so the analytic samples were 1,551 and 2,200 respectively.

### 2.6 Statistical analysis

Categorical variables are presented as frequencies and percentages, and composite scores and work-context items as means and standard deviations, with medians and interquartile ranges where distributions were skewed. Reporters and non-reporters were compared using the chi-square or Fisher's exact test for categorical variables and Welch's *t*-test for composite scores. Covariates were selected on prespecified clinical and organizational relevance, not bivariate significance. Model A estimated the adjusted odds of formally reporting at least one near-miss among the 1,551 observers, the only nurses with an opportunity to report. The prespecified parsimonious model was the principal specification; it included the four standardized domain indices, standardized time pressure and staffing adequacy, prior safety training, sex, employment status, OR assignment, weekly working hours, night shifts, surgical cases per day, and OR experience as the sole seniority measure, estimating 30 coefficients (approximately 16 events per parameter) with all variance inflation factors (VIFs) below 2.5. The fully adjusted model, which added age group, education, professional title, and total nursing experience (45 coefficients, approximately 11 events per parameter, and VIFs of 6.91 to 9.63 for these seniority variables), is reported as a sensitivity analysis without interpreting its covariate estimates. Model B estimated the adjusted odds of observing at least one near-miss in all 2,200 nurses using workload and process variables only: time pressure, staffing adequacy, OR nursing experience, OR specialty assignment, weekly working hours, night shifts per month, surgical case volume, and prior safety training. Model B is exploratory, and its estimates are described as factors associated with near-miss observation rather than predictors.

All reported estimates come from conventional maximum-likelihood logistic regression with Wald 95% CIs, with cluster-robust and mixed-effects models as sensitivity analyses, and all principal-model coefficients are reported, to three decimals where a confidence limit rounds to 1.00. The four domain indices were judged against a Bonferroni-adjusted threshold of *p* < 0.0125 (0.05/4), and estimates reaching only *p* < 0.05 are described as not robust to multiplicity. Reference categories were the largest category of each covariate in the principal model (female sex, permanent employment, general surgery, 41–48 weekly hours, 1–3 night shifts, 3–4 daily cases, and 1–5 years of OR experience). In Model B, general surgery and 41–48 weekly hours were also the references, whereas 1–2 daily cases, no night shifts, and 11–20 years of OR experience were retained so that higher workload and shorter experience are compared with a low-workload, experienced baseline; the fully adjusted model used chief nurse, 11–20 years of total and OR experience, cardiothoracic surgery, contract employment, and 1–2 daily cases. Because reference coding alters only covariate contrasts, it does not affect the domain estimates, model fit, or discrimination.

To account for clustering across 28 operating suites within 14 supervisory units (9 specialty teams), models were refitted using cluster-robust (sandwich) standard errors clustered by unit and as mixed-effects logistic regressions with a random intercept for supervisory unit. Supervisory units contained a median of 154 participants (range 98–217; IQR 131–178), and no unit contributed more than 9.9% of the sample. Because cluster-robust standard errors may be imprecise with 14 clusters, the mixed-effects model was fitted as a complement rather than a replacement. Calendar month of completion was added as a fixed effect in a sensitivity model, because four levels provide too little information to estimate a variance component reliably; a random intercept for month was fitted only as a secondary check. The intraclass correlation was estimated on the latent scale as the between-unit variance divided by the sum of that variance and π^2^/3 (39). Multicollinearity was assessed using VIFs, with values >5 flagged, and fit using the Hosmer-Lemeshow statistic.

Prespecified sensitivity and subgroup analyses comprised formal reporting modeled in all 2,200 nurses (a descriptive analysis that does not test the primary association), an outcome of at least two formal reports, separate exclusion of nurses with <12 months of OR experience and of trainee or temporary-contract nurses, stratification by professional title (junior: nurse and senior nurse; senior: supervisor, deputy chief, and chief nurse) and by prior safety training with product-term tests of heterogeneity, substitution of each reporting-system item for the index, and proportional-odds modeling of reported event volume. Further sensitivity analyses comprised the reporting-system index without the feedback item; the pre-report and post-report subscores entered together; the barrier index without the “no change” item; speaking-up and safety climate combined into one index and, separately, each domain index entered alone; and the complete-case analysis. Confidence intervals in subgroup analyses were not adjusted for multiplicity. All analyses were performed in R version 4.3 (R Foundation for Statistical Computing, Vienna, Austria) using the stats, MASS, lme4, sandwich, lmtest, lavaan, semTools, car, ResourceSelection, and pROC packages. Apart from the Bonferroni-adjusted threshold for the domain indices, a two-sided *p*-value <0.05 was considered statistically significant.

## 3 Results

### 3.1 Sample characteristics

The analytic sample comprised 2,200 OR nurses (Figure 1). Most participants were female (88.9%), 39.0% were aged 30–39 years, and 65.0% held a bachelor's degree. Nurses and senior nurses formed 65.5% of the workforce, 84.3% of participants were in permanent employment, and 58.1% had completed prior safety training. The largest groups worked 41–48 h per week (44.0%), managed 3–4 surgical cases per working day (48.2%), and had 1–5 years of OR experience (30.9%) (Table 1).

**Table 1:** Sociodemographic and occupational characteristics of participants (*N* = 2,200).

Characteristic	Category	No. (%)
Age group	<25 years	120 (5.5)
	25–29 years	500 (22.7)
	30–39 years	859 (39.0)
	40–49 years	553 (25.1)
	≥50 years	168 (7.6)
Sex	Female	1,956 (88.9)
	Male	244 (11.1)
Highest nursing education	Diploma	124 (5.6)
	Associate degree	501 (22.8)
	Bachelor's degree	1,431 (65.0)
	Master's degree or above	144 (6.5)
Professional title	Nurse	720 (32.7)
	Senior nurse	720 (32.7)
	Supervisor nurse	540 (24.5)
	Deputy chief nurse	165 (7.5)
	Chief nurse	55 (2.5)
Total nursing experience	<1 year	63 (2.9)
	1–5 years	567 (25.8)
	6–10 years	393 (17.9)
	11–20 years	788 (35.8)
	>20 years	389 (17.7)
OR nursing experience	<1 year	260 (11.8)
	1–5 years	679 (30.9)
	6–10 years	462 (21.0)
	11–20 years	560 (25.5)
	>20 years	239 (10.9)
Employment status	Permanent	1,855 (84.3)
	Contract	300 (13.6)
	Temporary	25 (1.1)
	Trainee	20 (0.9)
Main OR assignment	General surgery	582 (26.5)
	Orthopedics	414 (18.8)
	Obstetrics/gynecology	324 (14.7)
	Neurosurgery	165 (7.5)
	Cardiothoracic surgery	109 (5.0)
	Emergency OR	227 (10.3)
	Mixed/rotating OR	379 (17.2)
Weekly working hours	≤40 h	422 (19.2)
	41–48 h	967 (44.0)
	49–56 h	596 (27.1)
	>56 h	215 (9.8)
Night shifts per month	0	426 (19.4)
	1–3	931 (42.3)
	4–6	634 (28.8)
	≥7	209 (9.5)
Surgical cases per working day	1–2	279 (12.7)
	3–4	1,060 (48.2)
	5–6	658 (29.9)
	≥7	203 (9.2)
Previous safety training	No	922 (41.9)
	Yes	1,278 (58.1)

Percentages may not sum to 100 because of rounding. OR indicates operating room.

### 3.2 Near-miss burden and the reporting gap

Of the 2,200 nurses, 1,551 (70.5%) reported observing at least one perioperative near-miss during the preceding 12 months (Table 2; Figure 2A). Instrument, sponge, or needle count discrepancies were the most frequently observed subtype (47.3%), followed by patient identification or documentation errors (37.5%), medication, blood, or specimen-labeling failures (28.8%), sterility, infection control, or equipment failures (25.5%), and surgical site, procedure, or positioning events (15.5%) (Figure 2B); count discrepancies were the leading subtype in every professional title (Figure 2D). Among observers, 53.3% had observed 2–5 events and 2.8% more than 10.

**Table 2:** Near-miss observation, subtype distribution, and formal reporting.

Measure	Category	No. (%)
Observed ≥1 near-miss, full sample	Yes	1,551 (70.5)
	No	649 (29.5)
No. of near-misses observed, among observers	1	325 (21.0)
	2–5	827 (53.3)
	6–10	356 (23.0)
	>10	43 (2.8)
Near-miss subtype, among observers	Instrument/sponge/needle count	734 (47.3)
	Patient identification/documentation	581 (37.5)
	Medication/blood/specimen labeling	447 (28.8)
	Sterility/infection/equipment	396 (25.5)
	Site/procedure/positioning	240 (15.5)
Formally reported ≥1 near-miss, among observers	Yes	478 (30.8)
	No	1,073 (69.2)
No. of near-misses formally reported, among observers	1	198 (12.8)
	2–5	261 (16.8)
	6–10	19 (1.2)
	>10	0

Observation percentages use the full sample (*N* = 2,200); all other percentages use observers (*n* = 1,551). Respondents could report more than one subtype. The 69.2% of observers with no formal report is a nurse-level proportion, not the proportion of events unreported.

**Figure 2:**
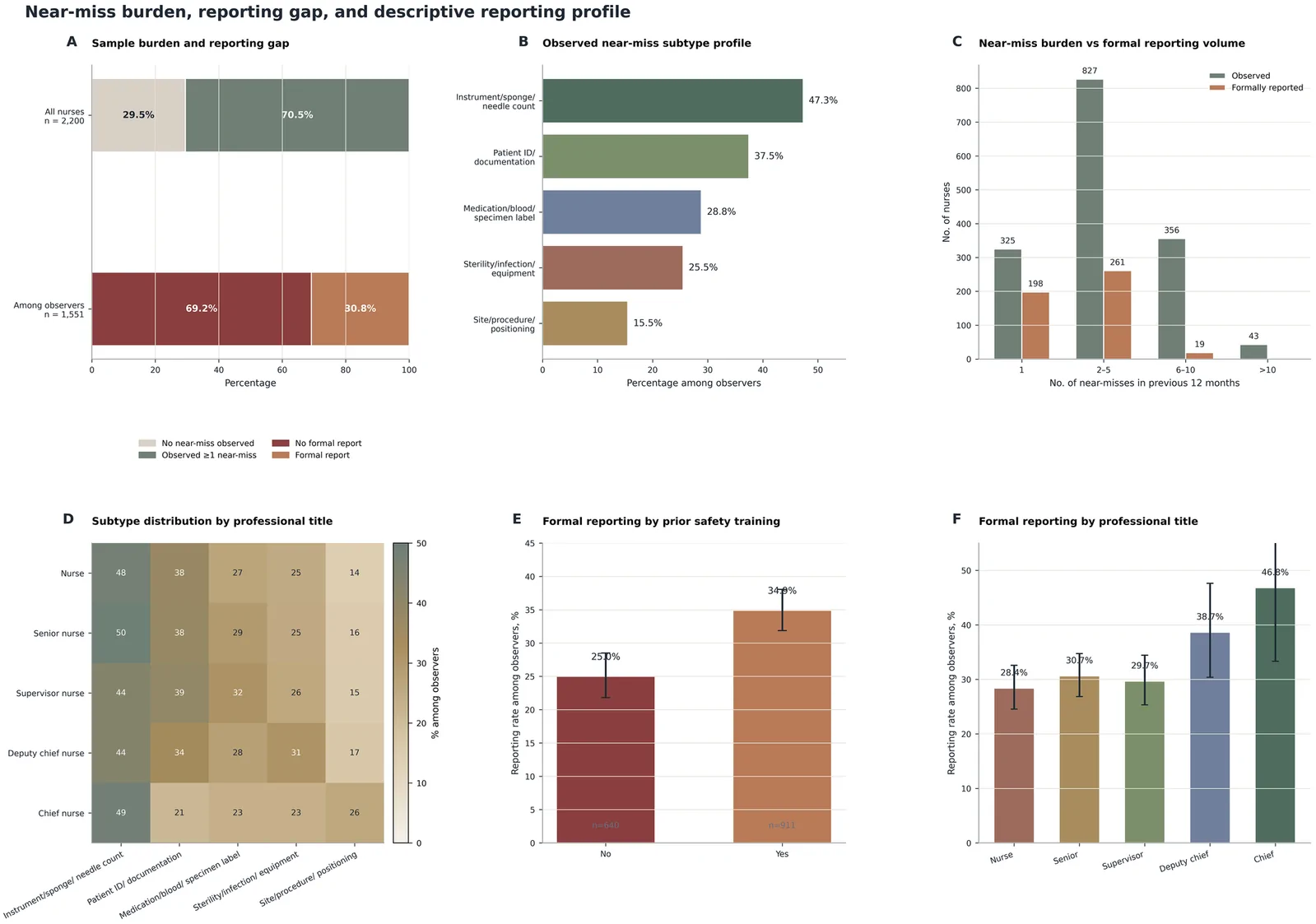
Near-miss burden, nurse-level reporting gap, and descriptive reporting profile. **(A)** Near-miss observation in the full sample (*N* = 2,200) and, among observers, the proportion making no formal report (nurse-level, not event-level). **(B)** Observed near-miss subtypes. **(C)** Observed vs. formally reported event volume by count band. **(D)** Subtype distribution by professional title. **(E)** Formal reporting by prior safety training. **(F)** Formal reporting by professional title. Panels B–F use near-miss observers (*n* = 1,551) as the denominator, and respondents could report more than one subtype. Error bars indicate 95% CIs where shown.

Of the 1,551 observers, 478 (30.8%) formally reported at least one event and 1,073 (69.2%) made no formal report of any near-miss during the preceding 12 months. This 69.2% is a nurse-level proportion of observers who never used the reporting system, not a proportion of near-miss events left unreported, because a nurse who observed several events and reported one was classified as a reporter. Reported counts were concentrated in the lowest bands: of 478 reporters, 198 (41.4%) reported a single event, 261 (54.6%) reported 2–5 events, and none reported more than 10 (Figure 2C). Formal reporting rates were higher among nurses with prior safety training than among those without training (34.9% vs. 25.0%) and increased across professional seniority, from 28.4% among nurses (30.7% among senior nurses, 29.7% among supervisor nurses, and 38.7% among deputy chief nurses) to 46.8% among chief nurses (Figures 2E,F).

### 3.3 Safety-culture domain properties and Likert distributions

Internal consistency was acceptable for speaking-up climate (Cronbach *α* = 0.831; *ω* = 0.836), reporting barriers (*α* = 0.801; *ω* = 0.807), and safety climate (*α* = 0.706; *ω* = 0.712), but lower for reporting-system function (*α* = 0.621; *ω* = 0.638). Four-domain model fit was marginal and did not meet conventional thresholds for CFI or TLI (CFI = 0.902, TLI = 0.888, RMSEA = 0.061, SRMR = 0.058). The HTMT ratio between speaking-up climate and safety climate was 0.87, exceeding the prespecified 0.85 threshold, so these indices were not treated as distinct constructs; they were strongly correlated (rho = 0.683), and both were inversely correlated with reporting barriers (rho = −0.649 and −0.523, respectively) (Table 3). Reporting barriers had the highest mean score (3.39, SD 0.78) and speaking-up climate the lowest (3.19, SD 0.91).

**Table 3:** Descriptive statistics, internal consistency, and inter-domain correlations.

Domain	Items	Mean (SD)	Median (IQR)	Range	Cronbach alpha	McDonald omega	Corrected item-total r
Reporting-system function	4	3.28 (0.78)	3.25 (2.75–3.75)	1.25–5.00	0.621	0.638	0.281 to 0.477
Speaking-up climate	6	3.19 (0.91)	3.17 (2.50–3.83)	1.00–5.00	0.831	0.836	0.413 to 0.664
Reporting barriers	7	3.39 (0.78)	3.43 (2.86–4.00)	1.00–5.00	0.801	0.807	0.440 to 0.665
Safety climate	3	3.29 (0.96)	3.33 (2.67–4.00)	1.00–5.00	0.706	0.712	0.503 to 0.540
Spearman correlation	Reporting-system function	Speaking-up climate	Reporting barriers	Safety climate			
Reporting-system function	1	0.613	−0.437	0.663			
Speaking-up climate	0.613	1	−0.649	0.683			
Reporting barriers	−0.437	−0.649	1	−0.523			
Safety climate	0.663	0.683	−0.523	1			

Domain scores are item means on a 1–5 scale; higher scores indicate better reporting-system function, a more favorable speaking-up or safety climate, or stronger reporting barriers. Confirmatory factor analysis: CFI 0.902, TLI 0.888, RMSEA 0.061, SRMR 0.058; heterotrait-monotrait ratio for speaking-up and safety climate, 0.87.

The most strongly endorsed barriers were fear of blame (63%), lack of time (57%), and fear of damaging relationships (56%) (Figure 3B), and the lowest-rated speaking-up items were questioning a surgeon (33%), questioning an anesthesiologist (35%), and speaking up to senior staff (37%) (Figure 3A). Among reporting-system and safety-climate items, agreement was highest for the belief that reporting prevents future harm (65%) and for knowing how to report (59%), and lowest for ease of reporting (36%), a fair, non-punitive response (36%), and feedback after reporting (23%), the lowest of all 20 items (Figure 3C).

**Figure 3:**
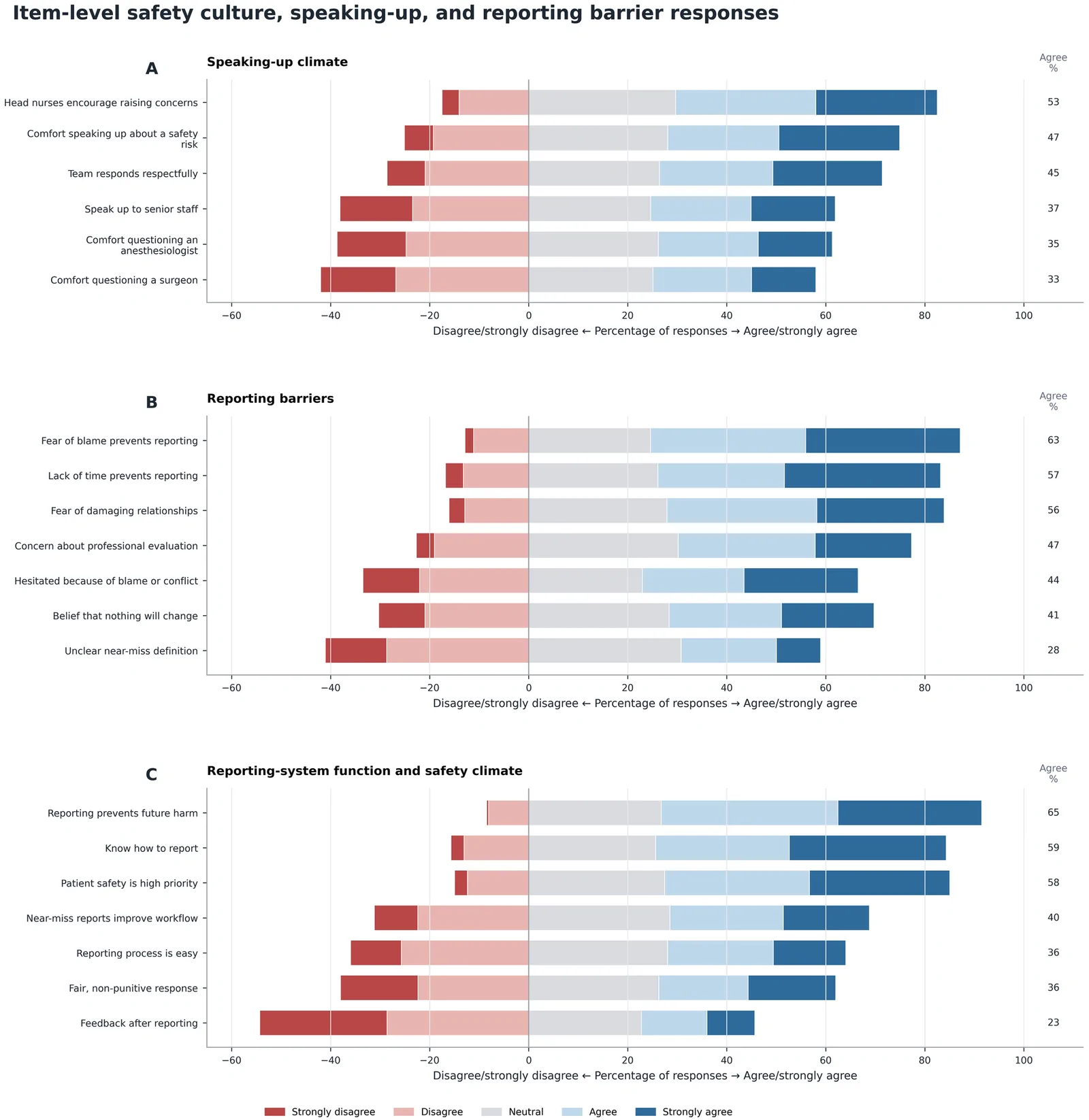
Item-level safety culture, speaking-up, and reporting-barrier responses (*N* = 2,200). **(A)** Speaking-up climate, **(B)** reporting barriers, and **(C)** reporting-system function and safety climate. Disagreement is plotted to the left of zero, and neutral, agree, and strongly agree responses to the right, in that order; items are ordered by agreement within each panel. The right-hand values indicate the percentage of respondents selecting “agree” or “strongly agree”; for reporting barriers, agreement indicates greater deterrence.

### 3.4 Bivariate comparisons between reporters and non-reporters

Among near-miss observers, formal reporters differed from non-reporters by sex, professional title, and prior safety training (all *p* < .05), but not by age group (*p* = .102) (Table 4). Reporters had higher reporting-system function (3.69 vs. 3.13), speaking-up climate (3.51 vs. 3.04), and safety climate scores (3.59 vs. 3.15), and lower reporting-barrier scores (3.12 vs. 3.56), all *p* < .001. Reporters also reported lower time pressure (3.99 vs. 4.24), better staffing adequacy (3.39 vs. 2.92), and more routine preoperative briefings (3.73 vs. 3.47) and postoperative debriefings (3.19 vs. 2.92), all *p* < .001. At least one domain item was missing for 36 of 478 reporters (7.5%) and 98 of 1,073 non-reporters (9.1%; *p* = .29), and the feedback item was missing for 0.8% and 2.3%, respectively (*p* = .047), indicating that non-reporters left this item unanswered more often.

**Table 4:** Bivariate comparisons of reporters and non-reporters among near-miss observers (*n* = 1,551).

Variable	Category or statistic	No report (*n* = 1,073)	Formal report (*n* = 478)	*p* value
Age group	<25 years	62 (5.8)	20 (4.2)	.102
	25–29 years	235 (21.9)	99 (20.7)	
	30–39 years	434 (40.4)	180 (37.7)	
	40–49 years	266 (24.8)	129 (27.0)	
	≥50 years	76 (7.1)	50 (10.5)	
Sex	Female	964 (89.8)	411 (86.0)	.027
	Male	109 (10.2)	67 (14.0)	
Professional title	Nurse	343 (32.0)	136 (28.5)	.031
	Senior nurse	362 (33.7)	160 (33.5)	
	Supervisor nurse	270 (25.2)	114 (23.8)	
	Deputy chief nurse	73 (6.8)	46 (9.6)	
	Chief nurse	25 (2.3)	22 (4.6)	
Previous safety training	No	480 (44.7)	160 (33.5)	<.001
	Yes	593 (55.3)	318 (66.5)	
Reporting-system function	Mean (SD)	3.13 (0.74)	3.69 (0.77)	<.001
Speaking-up climate	Mean (SD)	3.04 (0.88)	3.51 (0.88)	<.001
Reporting barriers	Mean (SD)	3.56 (0.74)	3.12 (0.78)	<.001
Safety climate	Mean (SD)	3.15 (0.93)	3.59 (0.93)	<.001
Time pressure	Mean (SD)	4.24 (0.94)	3.99 (1.06)	<.001
Staffing adequacy	Mean (SD)	2.92 (1.25)	3.39 (1.23)	<.001
Preoperative briefings routine	Mean (SD)	3.47 (1.11)	3.73 (1.02)	<.001
Postoperative debriefings routine	Mean (SD)	2.92 (1.15)	3.19 (1.17)	<.001
≥1 missing domain item	No. (%)	98 (9.1)	36 (7.5)	.29
Feedback item missing	No. (%)	25 (2.3)	4 (0.8)	.047

Categorical variables are No. (%) within reporting-status columns and continuous scores are mean (SD). *p* values are from chi-square tests (Fisher's exact test where expected counts were <5) and Welch's *t*-tests.

### 3.5 Adjusted associations With formal reporting: model A

In the principal parsimonious model among the 1,551 observers, reporting-system function showed the strongest association with formal reporting (aOR: 2.19 per 1-SD increase, 95% CI: 1.84–2.60; *p* < .001), and greater barrier burden was associated with lower odds of reporting (aOR: 0.67, 95% CI: 0.57–0.79; *p* < .001), both meeting the Bonferroni threshold (Table 5; Figure 4A). Speaking-up climate (aOR: 0.98, 95% CI: 0.81–1.19; *p* = .84) and safety climate (aOR: 0.84, 95% CI: 0.70–1.01; *p* = .07) were not associated with reporting after adjustment. In the fully adjusted model, safety climate had an aOR of 0.826 (95% CI: 0.684–0.998; *p* = .048), an upper limit that rounds to 1.00 and an estimate that does not meet the Bonferroni threshold and reverses the bivariate contrast. Time pressure, staffing adequacy, prior safety training, and the other covariates were not associated with reporting in the principal model, and the associations of male sex (aOR: 1.47) and 1–5 years of OR experience (aOR: 1.82) seen in the fully adjusted model did not persist. Discrimination was similar in the principal and fully adjusted models (AUC 0.741 and 0.746) (Figure 4C), and calibration of the principal model was adequate (Hosmer-Lemeshow *p* = .37).

**Table 5:** Adjusted associations with formal reporting among observers (Model A, principal model) and with near-miss observation in the full sample (Model B, exploratory).

Variable	Category	Model A, aOR (95% CI)	Model B, aOR (95% CI)
Reporting-system function	Per 1-SD increase	2.19 (1.84–2.60)	NA
Speaking-up climate	Per 1-SD increase	0.98 (0.81–1.19)	NA
Reporting barriers	Per 1-SD increase	0.67 (0.57–0.79)	NA
Safety climate	Per 1-SD increase	0.84 (0.70–1.01)	NA
Time pressure	Per 1-SD increase	0.91 (0.80–1.04)	1.14 (1.03–1.25)
Staffing adequacy	Per 1-SD increase	1.01 (0.87–1.17)	0.92 (0.83–1.01)
Previous safety training	Yes vs. no	1.06 (0.82–1.37)	1.02 (0.84–1.24)
Sex	Male vs. female	1.38 (0.96–1.98)	NA
Employment status	Permanent	1 [Reference]	NA
	Contract	0.94 (0.68–1.30)	NA
	Temporary	1.12 (0.41–3.05)	NA
	Trainee	0.87 (0.28–2.72)	NA
Main OR assignment	General surgery	1 [Reference]	1.06 (0.70–1.61)
	Orthopedics	1.08 (0.78–1.50)	0.97 (0.63–1.49)
	Obstetrics/gynecology	0.93 (0.65–1.33)	0.88 (0.57–1.36)
	Neurosurgery	1.15 (0.74–1.79)	1.12 (0.68–1.84)
	Cardiothoracic surgery	1.21 (0.74–1.98)	1 [Reference]
	Emergency OR	0.89 (0.59–1.34)	1.18 (0.74–1.88)
	Mixed/rotating OR	1.02 (0.72–1.44)	1.03 (0.67–1.58)
Weekly working hours	≤40 h	1.05 (0.76–1.45)	0.93 (0.73–1.18)
	41–48 h	1 [Reference]	1 [Reference]
	49–56 h	0.96 (0.73–1.26)	1.08 (0.86–1.35)
	>56 h	0.88 (0.58–1.33)	1.17 (0.84–1.63)
Night shifts per month	0	1.09 (0.79–1.50)	1 [Reference]
	1–3	1 [Reference]	1.04 (0.82–1.32)
	4–6	0.97 (0.74–1.27)	1.11 (0.85–1.44)
	≥7	0.92 (0.60–1.41)	1.21 (0.85–1.72)
Surgical cases per day	1–2	0.81 (0.55–1.19)	1 [Reference]
	3–4	1 [Reference]	0.84 (0.63–1.12)
	5–6	1.19 (0.91–1.56)	0.68 (0.49–0.93)
	≥7	0.93 (0.61–1.42)	0.63 (0.42–0.94)
OR nursing experience	<1 year	0.98 (0.60–1.60)	0.53 (0.35–0.80)
	1–5 years	1 [Reference]	0.75 (0.53–1.06)
	6–10 years	0.80 (0.58–1.10)	0.69 (0.52–0.92)
	11–20 years	0.71 (0.49–1.02)	1 [Reference]
	>20 years	0.72 (0.46–1.13)	1.09 (0.74–1.62)
Model fit	No. (events)	1,551 (478)	2,200 (1,551)
	AUC	0.741	0.600
	Hosmer-Lemeshow	*Χ*^2^ = 8.72, *p* = .37	*χ*^2^ = 11.62, *p* = .17

Estimates are from conventional maximum-likelihood logistic regression with Wald 95% CIs. Model A is the principal parsimonious model (30 coefficients); Model B is exploratory. Domain and work-context scores are standardized, so aORs are per 1-SD increase. Reporting-system function and reporting barriers met the Bonferroni-adjusted threshold (*p* < .0125). Reference categories are shown for each model. Fully adjusted model estimates are given in Table 6.

aOR indicates adjusted odds ratio; AUC, area under the receiver operating characteristic curve; NA, not included in the model; OR, operating room.

**Figure 4:**
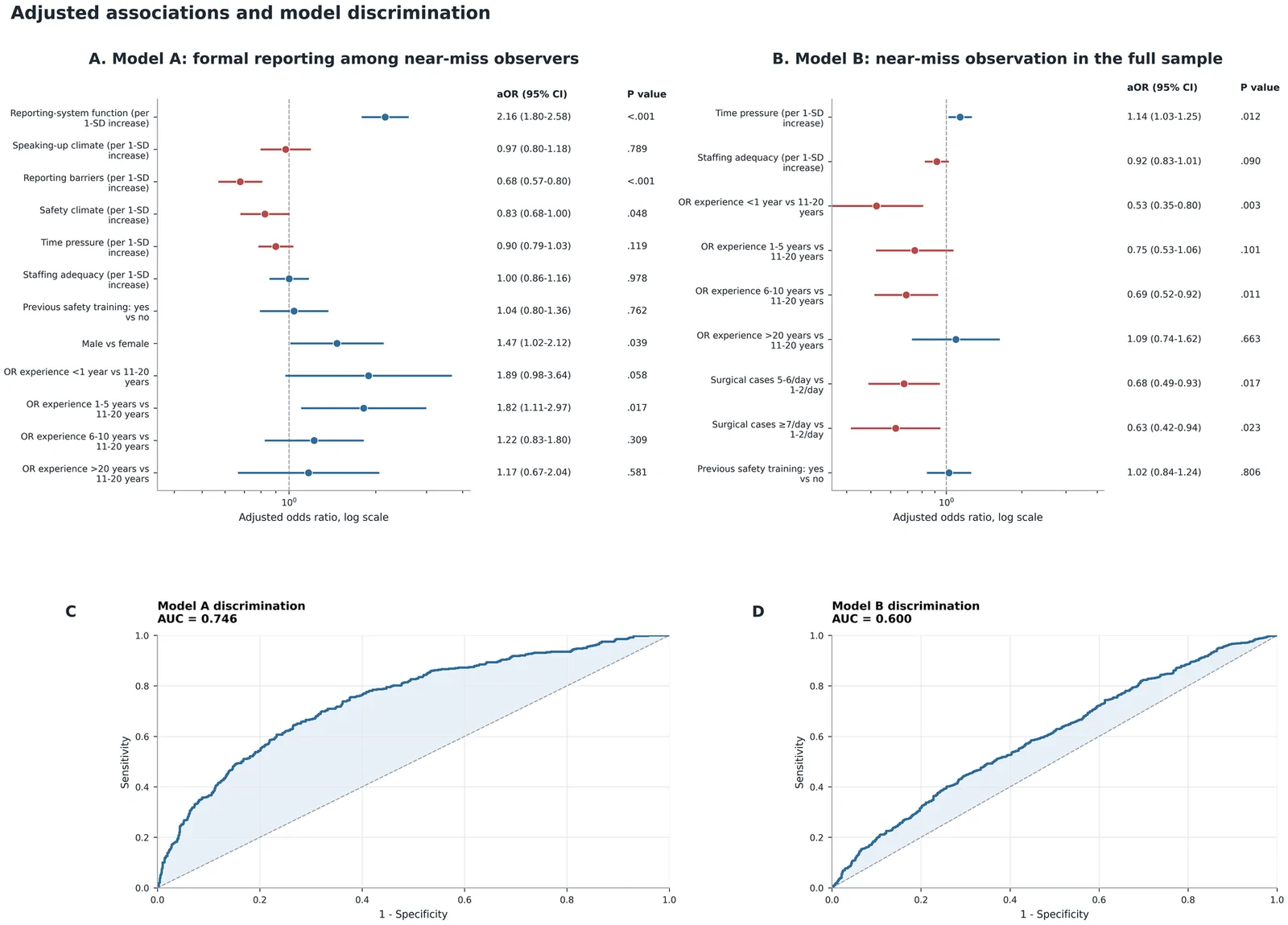
Adjusted associations and model discrimination. **(A)** Adjusted associations with formal reporting among near-miss observers (Model A, principal model; *n* = 1,551). **(B)** Factors associated with near-miss observation in the full sample (Model B, exploratory; *N* = 2,200). **(C)** Receiver operating characteristic curve for Model A. **(D)** Receiver operating characteristic curve for Model B. Points represent adjusted odds ratios, horizontal bars represent 95% confidence intervals, and odds ratios are plotted on a log scale. Domain and work-context estimates are per 1-SD increase, and the dashed line marks an odds ratio of 1. AUC indicates area under the receiver operating characteristic curve; OR, operating room.

Each reporting-system item substituted for the index gave concordant adjusted associations per 1-SD increase: 1.61 (95% CI: 1.39–1.87) for knowing how to report, 1.71 (1.48–1.98) for ease of submission, 1.94 (1.67–2.25) for receipt of feedback, and 1.78 (1.53–2.07) for belief that reporting prevents harm, with overlapping intervals. The two items that do not depend on having reported were each associated with reporting, and feedback, the item most likely to reflect earlier reporting, was numerically strongest.

### 3.6 Factors associated with near-miss observation: model B (exploratory)

In the exploratory full-sample model, higher time pressure was associated with greater odds of observing a near-miss (aOR: 1.14, 95% CI: 1.03–1.25; *p* = .012) (Table 5; Figure 4B). Compared with 1–2 cases per day, 5–6 cases (aOR: 0.68, 95% CI: 0.49–0.93) and ≥7 cases per day (aOR: 0.63, 95% CI: 0.42–0.94) were associated with lower odds of reported observation, as were <1 year (aOR: 0.53, 95% CI: 0.35–0.80) and 6–10 years (aOR: 0.69, 95% CI: 0.52–0.92) of OR experience compared with 11–20 years. Staffing adequacy (aOR: 0.92, 95% CI: 0.83–1.01) and prior safety training (aOR: 1.02, 95% CI: 0.84–1.24) were not associated with observation. Model B discrimination was only marginally above chance (AUC 0.600; Figure 4D), despite adequate calibration (Hosmer-Lemeshow *p* = .17), so the measured variables explain little of the between-nurse variation in observation and these associations are hypothesis-generating.

### 3.7 Sensitivity and subgroup analyses

Across the prespecified sensitivity and subgroup analyses, excluding the descriptive whole-workforce model, aORs ranged from 1.97 to 2.41 for reporting-system function and from 0.63 to 0.76 for reporting barriers (Table 6). Adding calendar month as a fixed effect (2.14, 95% CI: 1.79–2.56; 0.69, 0.58–0.82), cluster-robust errors across the 14 supervisory units (2.16, 1.76–2.65; 0.68, 0.55–0.83), a random intercept for unit (ICC = 0.019), or a random intercept for month (2.15, 1.77–2.61; 0.69, 0.58–0.83) left both associations unchanged, as did redefining the outcome as at least two formal reports (2.19; 0.74) and excluding nurses with <12 months of OR experience (2.12; 0.65) or trainee and temporary staff (2.21; 0.68). Associations were directionally consistent in junior (2.35) and senior (1.97) title subgroups and in nurses without (2.41) and with (2.12) prior safety training, and the descriptive whole-workforce model, which does not test the primary association, gave estimates of 2.14 and 0.78. Ordinal modeling of reported event volume gave concordant results (OR 2.08, 95% CI: 1.78–2.44; and 0.71, 0.61–0.83), so the findings did not depend on dichotomization. No interactions were detected by professional title or prior safety training (*p* = .38 and *p* = .21, respectively).

**Table 6:** Sensitivity and subgroup analyses of formal reporting Among observers.

Analysis	*N*	Events	Reporting-system function, aOR (95% CI)	Reporting barriers, aOR (95% CI)	Speaking-up climate, aOR (95% CI)	Safety climate, aOR (95% CI)	AUC
Principal parsimonious model	1,551	478	2.19 (1.84–2.60)	0.67 (0.57–0.79)	0.98 (0.81–1.19)	0.84 (0.70–1.01)	0.741
Fully adjusted model	1,551	478	2.16 (1.80–2.58)	0.68 (0.57–0.80)	0.97 (0.80–1.18)	0.826 (0.684–0.998)	0.746
Whole-workforce reporting model (descriptive)	2,200	478	2.14 (1.81–2.53)	0.78 (0.67–0.91)	0.97 (0.82–1.16)	0.84 (0.70–1.00)	0.722
Outcome: ≥2 formal reports	1,551	280	2.19 (1.75–2.74)	0.74 (0.60–0.91)	1.13 (0.89–1.44)	1.22 (0.96–1.55)	0.807
Excluding <1 year of OR experience	1,392	429	2.12 (1.75–2.56)	0.65 (0.55–0.78)	0.92 (0.75–1.13)	0.87 (0.71–1.06)	0.749
Excluding trainee or temporary staff	1,522	468	2.21 (1.84–2.65)	0.68 (0.57–0.80)	0.97 (0.80–1.18)	0.81 (0.67–0.98)	0.746
Junior title subgroup	1,001	296	2.35 (1.87–2.97)	0.65 (0.53–0.80)	0.91 (0.71–1.17)	0.79 (0.62–1.01)	0.748
Senior title subgroup	550	182	1.97 (1.44–2.70)	0.76 (0.56–1.01)	1.10 (0.80–1.52)	0.89 (0.64–1.24)	0.771
No prior safety training	640	160	2.41 (1.77–3.30)	0.74 (0.56–0.99)	1.04 (0.74–1.45)	0.63 (0.46–0.86)	0.743
Prior safety training	911	318	2.12 (1.68–2.68)	0.63 (0.50–0.79)	0.90 (0.70–1.15)	0.95 (0.75–1.22)	0.773
Calendar month as fixed effect	1,551	478	2.14 (1.79–2.56)	0.69 (0.58–0.82)	NA	NA	NA
Cluster-robust errors by supervisory unit	1,551	478	2.16 (1.76–2.65)	0.68 (0.55–0.83)	NA	NA	NA
Random intercept for unit (ICC 0.019)	1,551	478	2.17 (1.80–2.61)	0.68 (0.57–0.81)	NA	NA	NA
Random intercept for month	1,551	478	2.15 (1.77–2.61)	0.69 (0.58–0.83)	NA	NA	NA
Proportional-odds model, reported event volume	1,551	478	2.08 (1.78–2.44)	0.71 (0.61–0.83)	NA	NA	NA
Item substitution for the index	1,551	478	Know how 1.61 (1.39–1.87); ease 1.71 (1.48–1.98); feedback 1.94 (1.67–2.25); prevents harm 1.78 (1.53–2.07)	NA	NA	NA	NA
Index without the feedback item	1,551	478	1.92 (1.62–2.28)	0.69 (0.58–0.81)	1.00 (0.83–1.21)	0.86 (0.71–1.03)	0.731
Pre-report and post-report subscores entered together	1,551	478	Pre-report 1.54 (1.31–1.81); post-report 1.63 (1.38–1.93)	0.68 (0.58–0.81)	0.99 (0.82–1.20)	0.85 (0.70–1.02)	0.744
Barrier index without the “no change” item	1,551	478	2.20 (1.85–2.62)	0.70 (0.59–0.82)	0.99 (0.82–1.20)	0.84 (0.70–1.01)	0.740
Combined speaking-up and safety climate index	1,551	478	2.21 (1.86–2.63)	0.67 (0.57–0.79)	Combined index 0.90 (0.76–1.07)	NA	0.740
Each domain entered alone	1,551	478	2.41 (2.08–2.80)	0.55 (0.48–0.63)	1.62 (1.42–1.85)	1.59 (1.39–1.81)	NA
Complete-case analysis	1,417	442	2.17 (1.80–2.62)	0.67 (0.56–0.80)	0.99 (0.81–1.21)	0.85 (0.70–1.03)	0.743

Rows from the whole-workforce model through the proportional-odds model use the fully adjusted covariate set; the item-substitution row and the last five rows use the principal model. All estimates are per 1-SD increase from conventional logistic regression with Wald 95% CIs unless stated; the proportional-odds row reports odds ratios. Interaction *p* values: professional title, .38; prior safety training, .21. Subgroup CIs are not adjusted for multiplicity. The whole-workforce model includes 649 nurses who observed no near miss, combines non-observation with non-reporting, and does not test the primary association. aOR indicates adjusted odds ratio; AUC, area under the receiver operating characteristic curve; ICC, intraclass correlation coefficient; NA, not applicable or not estimated; OR, operating room.

The reporting-system index without the feedback item remained associated with reporting (aOR: 1.92, 95% CI: 1.62–2.28), and when entered together, both the pre-report accessibility subscore (aOR: 1.54, 95% CI: 1.31–1.81) and the post-report experience subscore (aOR: 1.63, 95% CI: 1.38–1.93) were associated with reporting. Removing the “no change” item left the barrier association unchanged (aOR: 0.70, 95% CI: 0.59–0.82). A combined climate index replacing speaking-up and safety climate was not associated with reporting (aOR: 0.90, 95% CI: 0.76–1.07), whereas each domain entered alone showed its bivariate direction, including speaking-up climate (aOR: 1.62, 95% CI: 1.42–1.85) and safety climate (aOR: 1.59, 95% CI: 1.39–1.81), so their adjusted estimates reflect mutual adjustment. The complete-case analysis (*n* = 1,417; 442 reporters) reproduced the principal estimates (aOR: 2.17, 95% CI: 1.80–2.62; and 0.67, 0.56–0.80).

## 4 Discussion

This cross-sectional study of 2,200 OR nurses at a two-campus tertiary hospital identified a substantial burden of perioperative near-miss exposure but a large gap between observation and formal reporting. More than two-thirds of participants reported observing at least one near-miss event during the preceding 12 months, yet fewer than one-third of observers formally reported an event; this is a nurse-level gap, meaning that 69.2% of observers never used the reporting system, not that 69.2% of events went unreported. Instrument, sponge, or needle count discrepancies were the most frequent near-miss subtype, followed by patient identification or documentation errors, medication or specimen-labeling failures, sterility or equipment failures, and surgical site, procedure, or positioning events. Formal reporting was most strongly associated with perceived reporting-system function and reporting barriers, whereas speaking-up and safety climate were not independently associated with reporting once these overlapping domains were mutually adjusted. These associations are consistent with, but cannot establish, a view in which reporting depends less on individual willingness than on whether nurses perceive the reporting pathway as accessible, responsive, and non-punitive (18, 19).

The high frequency of near-miss observation in this study is consistent with the intrinsic risk profile of the operating room, where complex workflows, time-critical decisions, multidisciplinary team interactions, and repeated handovers create multiple opportunities for latent failures. The predominance of instrument, sponge, and needle count discrepancies is clinically plausible and aligns with surgical safety literature showing that count discrepancies and retained surgical item risks remain persistent perioperative hazards. Takahashi and colleagues reported 106 near-miss events involving retained foreign bodies among 49,831 operations, corresponding to 2.13 near-misses per 1,000 operations, while Greenberg and colleagues reported that intraoperative count discrepancies occurred in approximately one in eight surgical cases and most often involved sponges, instruments, or needles (13, 40). This pattern also accords with Chinese operating room evidence from Song and colleagues, who reported a 27.7% prevalence of self-reported medical errors among operating room nurses, with surgical-instrument-related errors as the most frequent subtype (14). Together, these findings support the interpretation that count-related near-misses are not peripheral administrative events but central indicators of perioperative system reliability. Because subtypes were recorded only for observed events and not for reported events, we cannot determine whether count discrepancies or any other subtype were disproportionately absent from the formal reporting system.

The reporting gap observed in this study is also consistent with the broader patient-safety literature, in which voluntary reporting systems capture only a fraction of safety events. Although near misses are valuable because they provide information about system weaknesses before patient harm occurs, they are especially vulnerable to under-reporting because they may be resolved informally, perceived as minor, or excluded from formal documentation once harm is avoided. Previous Chinese studies have similarly shown that safety culture and management response are important correlates of reporting intention and reporting behavior (41, 42). The present finding that only 30.8% of near-miss observers formally reported any event therefore reinforces the concept that near-miss surveillance cannot depend solely on individual vigilance; it requires a reporting architecture that converts frontline detection into organizational learning. Because event counts were captured in ordered bands rather than as exact counts, the proportion of individual events that entered the reporting system cannot be estimated from these data, and no event-level reporting fraction is claimed here.

Reporting-system function showed the strongest adjusted association with formal reporting, each 1-SD increase corresponding to more than twice the adjusted odds of reporting. Two of its four items, receipt of feedback after reporting and the belief that reporting prevents harm, can be formed largely through a nurse's own earlier reports, so their association with reporting is partly built into the measure and may reflect the consequences of reporting rather than its antecedents. Consistent with this, the feedback item had the lowest agreement of all 20 items, was missing more often among non-reporters, and showed the numerically strongest item-level association. The two items that do not depend on having reported, knowing how to report and ease of submission, were nevertheless each associated with reporting, the index remained associated after the feedback item was removed, and the pre-report accessibility subscore remained associated when entered alongside the post-report experience subscore. The more defensible interpretation is therefore an association between reporting and the perceived accessibility and usability of the reporting pathway, with the contribution of feedback open to reverse direction. The index also had the lowest internal consistency of the four domains (alpha 0.621, omega 0.638), so it is best understood as a pragmatic summary of these perceptions rather than a validated construct. The finding is consistent with, but does not establish, a modifiable organizational target (43, 44). A functional reporting system is not merely a technical platform; it includes clear definitions, accessible procedures, timely acknowledgement, feedback to the reporter, and evidence that reported events lead to corrective action. This interpretation is supported by Chinese patient-safety culture evidence, in which event reporting was among the lowest-scoring domains, indicating that healthcare workers may believe in improvement while still perceiving formal reporting as weak or difficult (5). Thus, the present results suggest that OR nurses may be more willing to report when the reporting system is visible, simple, and responsive, and when reporting is linked to feedback rather than administrative burden.

The inverse association between reporting barriers and formal reporting was expected and remained robust across sensitivity analyses, including removal of the item stating that reporting produces no change, which may itself reflect earlier reporting experience. Fear of blame, lack of time, and fear of damaging professional relationships were the most strongly endorsed barriers, which is consistent with international and Chinese evidence that punitive response, legal anxiety, workload, and relational consequences are associated with lower reporting (42, 45, 46). These findings are particularly important in the OR because near-miss reporting often requires nurses to document events involving surgeons, anesthesiologists, instruments, specimens, or procedural flow, all of which may be embedded within hierarchical and interprofessional relationships (47, 48). The data therefore support a shift from exhorting nurses to “report more” toward redesigning the social and procedural conditions under which reporting occurs.

The attenuation of speaking-up climate after adjustment deserves careful interpretation. In bivariate analysis, reporters had substantially higher speaking-up climate scores than non-reporters, but this association disappeared after adjustment for reporting-system function, barrier burden, and safety climate. The defensible reading of this result is statistical rather than substantive: after adjustment for several strongly correlated organizational indices, the climate score added no independent information about the outcome. It does not follow that speaking up is unimportant, and our design cannot address that question. Speaking-up climate as measured here is a generalized perception, whereas speaking up as studied behaviorally is an event-based interaction whose occurrence and consequences depend on its form and on the recipient's response (22–26). Speaking-up research also shows that healthcare workers are more likely to voice concerns when supervisors invite input, respond constructively, and create psychological safety, whereas unpredictable or dismissive responses inhibit voice (21, 49, 50). Within the sequence from recognition to organizational learning, speaking-up climate bears mainly on real-time voice, which precedes the decision to file a report; adjusting for barriers and reporting-system function, which sit closer to that decision, may absorb part of its association, so the null adjusted estimate should not be read as the absence of an effect.

The item-level speaking-up findings further highlight the role of hierarchy in perioperative safety. Only one-third of nurses agreed that they were comfortable questioning a surgeon, and similarly low proportions reported comfort questioning an anesthesiologist or senior staff member. These results are consistent with evidence that hierarchical gradients strongly influence safety voice, especially when the concern involves a senior clinician or interprofessional authority (21, 29). In Chinese hospitals, where professional role boundaries and seniority norms may be more pronounced, nurses may be especially reluctant to challenge physicians or senior staff unless institutional norms explicitly authorize and protect safety voice. Consequently, OR safety interventions should address hierarchy directly through team briefings, closed-loop communication, escalation protocols, and leadership behaviors that normalize respectful challenge (51, 52).

The association between safety climate and formal reporting was complex. Although reporters had higher unadjusted safety-climate scores, the fully adjusted association was inverse (aOR: 0.83), with an upper confidence limit that rounds to unity; it did not meet the Bonferroni threshold, was not present in the principal model, and reversed a strong bivariate contrast in the presence of correlations above 0.65 with two co-modeled domains. Safety climate and speaking-up climate did not achieve discriminant separation, and when they were combined into one index the association was null, whereas each entered alone was positively associated with reporting. This pattern indicates that the inverse coefficient reflects mutual adjustment of overlapping constructs rather than an adverse effect of a favorable climate. Broad safety-culture domains often overlap with proximal factors such as feedback, non-punitive response, communication openness, and management support, as reflected in instruments such as the Safety Attitudes Questionnaire and HSOPSC (35, 46, 53). Because safety climate may act on reporting through speaking-up climate and reporting barriers, adjusting for these intermediate factors may also amount to over-adjustment, so the residual coefficient does not represent its total association with reporting.

Prior safety training was associated with higher reporting in unadjusted comparisons but not after adjustment, and no interaction by training was detected. Training may therefore improve recognition and procedural knowledge without offsetting the associations of system function or perceived barriers, consistent with evidence that a non-punitive culture and feedback after reporting are also needed for sustained reporting (5, 51).

Unadjusted reporting rose across professional seniority, from 28.4% among nurses to 46.8% among chief nurses, which may reflect greater authority, institutional knowledge, and protection from relational consequences among senior staff. Seniority and experience estimates in the fully adjusted model arose from a collinear covariate cluster and did not persist in the principal model, so they are not interpreted, and qualitative work is needed to establish whether and how position influences reporting (5, 19, 42).

Factors associated with near-miss observation differed from those associated with formal reporting, supporting the distinction between event recognition and reporting behavior, although Model B discriminated only marginally above chance and these associations are exploratory. Time pressure was associated with greater odds of observing a near-miss, consistent with the view that workload increases opportunities for error. Conversely, higher daily surgical volume and less than one year of OR experience were associated with lower reported observation. Although counterintuitive, this may reflect reduced attention to low-salience deviations, greater reliance on informal correction, or differences in what nurses recognize and later remember: experienced nurses may identify more latent hazards and classify corrected deviations as near misses, whereas novices may not recognize them or may recall only salient events. This interpretation is consistent with human-factors models in which safety-event detection depends on exposure, vigilance, expertise, situational awareness, and local norms for defining deviations (54, 55).

These associations suggest, without establishing, implications for perioperative safety governance that can be expressed as five operational steps. First, make reporting easy through a short point-of-care form with minimal free text. Second, define what should be reported, using short OR-specific examples such as count discrepancies, labeling errors, and identification mismatches, since more than a quarter of nurses (28%) found the near-miss definition unclear. Third, protect the reporter through just-culture policies that distinguish human error, at-risk behavior, and reckless behavior (56, 57). Fourth, close the feedback loop by acknowledging every report and communicating the action taken, which addresses the item with the lowest agreement in this study. Fifth, demonstrate organizational learning by showing staff examples in which reports changed workflow, equipment, staffing, training, or policy, and by reviewing near misses in routine team debriefings (58–61). Treated as a learning system rather than a disciplinary archive, near-miss data are most valuable when they reveal recurrent process vulnerabilities before harm occurs, and should be integrated into institutional quality dashboards, morbidity and mortality review, infection-control surveillance, instrument-count governance, and nursing-sensitive quality indicators (35).

This study has several limitations. First, the design is cross-sectional with concurrent self-report, so direction cannot be established, as prior reporting and feedback may have improved perceptions of the system rather than the reverse, and common-method variance may inflate associations because a nurse with positive views of the organization may rate every domain favorably and also report more; anonymity and item-level analyses reduce but do not remove this risk. Second, 12-month recall invites recall bias, telescoping, selective recall of serious events, and confusion of near misses with corrected deviations, which may differ by experience, and these self-reports could not be verified. Third, responses were not linked to objective data such as reporting volume, feedback rates, corrective actions, repeat incidents, case mix, staffing, overtime, or count discrepancies. Fourth, the outcome was participant-level: 69.2% denotes observers who filed no report, not unreported events, banded counts preclude an event-level fraction, and subtype was recorded for observed events only. Fifth, the four composites are pragmatic indices rather than validated constructs, built from adapted and newly written items pretested in only five nurses, with low internal consistency for reporting-system function (alpha 0.621, omega 0.638), marginal confirmatory fit, and poor discrimination between speaking-up and safety climate; the feedback item was also missing more often among non-reporters, and the complete-case and feedback-free analyses address but cannot exclude its outcome dependence. Sixth, seniority variables were collinear in the fully adjusted model, so covariate inference rests on the parsimonious model. Seventh, specialty complexity, case acuity, team familiarity, and workflow interruptions were not measured, which may explain the weak discrimination of the observation model. Eighth, the 331 nonrespondents were not compared with respondents, so participation may have favored nurses with stronger views. Ninth, findings from one two-campus tertiary institution over four months may not generalize to other governance, staffing, or reporting contexts or reflect seasonal variation. Finally, whether reports produced corrective action or sustained risk reduction could not be determined; longitudinal mixed-methods work linking survey and reporting-system data is needed.

## 5 Conclusion

Perioperative near misses were commonly observed by operating room nurses in this two-campus institution, yet most observers made no formal report of any event during the preceding 12 months; this gap describes nurses who never used the reporting system, not a count of unreported events. Count discrepancies were the most frequent near miss. Formal reporting was most strongly associated with perceived reporting-system function and barrier burden rather than with speaking-up climate. Because perceptions of feedback may follow reporting, the most defensible correlate is how accessible and easy nurses find the reporting pathway. These cross-sectional, self-reported associations cannot establish direction. Hospitals may reasonably test a short point-of-care report form, OR-specific examples of reportable events, just-culture protection for reporters, acknowledgment and feedback on every report, and visible examples of changes made from reports; prospective evaluation is needed to establish whether such changes alter reporting behavior.

## Data Availability

The raw data supporting the conclusions of this article will be made available by the authors, without undue reservation.
